# MEASURING AND MANIPULATING YOUTHFUL FACTORS IN EXTRACELLULAR VESICLES THROUGH ENGINEERING AND EXERCISE

**DOI:** 10.1093/geroni/igae098.0738

**Published:** 2024-12-31

**Authors:** Fabrisia Ambrosio

**Affiliations:** Harvard Medical School, Boston, Massachusetts, United States

## Abstract

Numerous studies employing heterochronic blood exchange have demonstrated the crucial age-dependent role of circulating factors in regulating the function of multiple tissues. To date, investigations into the mechanisms underlying the beneficial effect of young blood on healing cascades have primarily focused on circulating proteins. However, accumulating evidence suggests that circulating extracellular vesicles (EVs) may play a previously unappreciated role in the regulation of tissue health. EVs are membrane-enclosed paracrine mediators that traffic between anatomically remote sites to regulate physiological function and pathologic processes in target cells. A better understanding of the molecular mechanisms guiding age-related alterations in EV cargoes may pave the way towards the development of novel therapeutics that can promote a more youthful phenotype in older individuals. In her presentation, Dr. Ambrosio will summarize recent work from her laboratory identifying an age-related disruption in the transfer of information from circulating EVs to target cells and the impact of this this disrupted communication flow on tissue maintenance and regenerative potential over time. Towards the development of novel EV-based therapeutic strategies to counteract these declines, Dr. Ambrosio will share ongoing studies employing engineering and rehabilitative strategies designed to promote tissue function in an elderly population.

